# The benefit of cement-augmented pedicle screws for revision surgery in osteoporotic bone – a cadaveric study

**DOI:** 10.1186/s12891-026-09971-y

**Published:** 2026-05-14

**Authors:** K. Kafchitsas, G. Kontovazainitis, P. Diaremes, P. Drees, S. Winkler

**Affiliations:** 1 Department of Orthopaedics and Traumatology, University Medical Center of the Johannes Gutenberg University, 55131 Mainz, Germany; 2 Practice of Periodontology & Implant Dentistry, Athens, Greece; 3 Clinic for Trauma Surgery and Orthopaedics, Goethe University Hospital Frankfurt, Frankfurt, Germany; 4 Asklepios Orthopaedic Hospital Lindenlohe, Lindenlohe, Germany

**Keywords:** Cadaveric bone, Cement-augmented screw, Osteoporotic bone, Pull-out test

## Abstract

**Purpose:**

Increasing numbers of spine surgery cases and an aging society led to challenges during revision surgery with osteoporotic bone. Safe and stable operative techniques are required.

**Methods:**

In this ex vivo study, human lumbar vertebrae were prepared and then screened for assessing bone density. Eighteen donors (65 vertebrae) with osteoporotic conditions were included. Primary dorsal instrumentation was performed using either standard pedicle screws (6.35 mm diameter; Group A, *n* = 50) or vertebroplasty screws (VT, 6.35 mm; Group B, *n* = 15).

This was followed by a standardized pull-out test cycle, after which the vertebrae of group A exhibited pedicle perforations replicating revision conditions. Subsequently, 50 vertebrae from Group A were reassigned to simulate revision conditions and divided into two subgroups (n = 25 each): Group A1 received conventional pedicle screws with an increased diameter (7.5 mm), while Group A2 was treated with cement-augmented pedicle screws (VT, 6.35 mm). A second pull-out test was performed, and statistical analysis was conducted.

**Results:**

The specimens with conventional screws had a lower mean pull-out strength compared to primarily cement-augmented screws during the first pull-out cycle 662.8 ± 365.1 N vs. 1066.1 ± 213.7 N. In the revision model, cement-augmented screws (Group A2) demonstrated a significant higher median pull-out strength compared to larger-diameter conventional screws (Group A1) (687.5 N [IQR: 448.8–1137.5 N]; vs. 486.5 N [IQR: 338.8–605.0 N]).

**Conclusion:**

In this ex vivo revision model of osteoporotic bone, increasing screw diameter alone resulted in reduced pull-out strength compared to primary instrumentation, whereas cement-augmented pedicle screws consistently demonstrated superior fixation in both test cycles. These findings highlight the limited effectiveness of increasing screw diameter under revision conditions and support cement augmentation as a biomechanically advantageous strategy in osteoporotic bone, while clinical validation remains necessary.

## Introduction

Osteoporosis increases the risk for osteoporotic vertebral compression fractures (OCF). Untreated or conservative treatment of OCFs can have damaging long-term effects. Early surgical treatment with percutaneous vertebral augmentation (PVA) seems to offer higher benefits and lower morbidity [1]. Osteoporosis is associated with elevated complication rates after primary spine surgery [2] and increases the risk for revisions [3].

After removal of loosened pedicle screws, the surgeon can either reinsert a thicker pedicle screw or extend the construct to the adjacent level.

In revision surgery, the newly inserted pedicle screws need to have firm placement in the osteoporotic vertebrae. The following study was designed to examine two possible surgical options in case of revision surgery of osteoporotic bone: either by inserting a conventional pedicle screw with a larger diameter or by using a cement-augmented pedicle screw. We hypothesised that the second option offers higher stability and a lower pull-out risk without requiring extension the spondylodesis construct.

## Material and method

### Patients and ethics

We used 80 human vertebrae from the lumbar spine of 18 donors, eight women and ten men. The specimens were prepared and processed according to standards by Wilke et al. [4] post mortem by the anatomic institute of the university of Vienna, Austria. Before their passing, all the participants had provided informed consent. Ethics committee of the Medical Faculty (University of Frankfurt am Main, Germany) approval was waived as this cadaver study involved specimens with completely anonymized data from a body donor program. All individuals have provided informed consent prior to their death to be used for medical research or education. All methods were carried out in accordance with local guidelines and regulations (Instituto de Biomecánica (IBV), Camino de Vera s/n, Edificio 9 C, 46022 · Valencia Spain).

Mean age of the patients was 73 years (min. 63 - max. 97 years). Radiologic examination of each specimen was performed to rule out traumatic or metastatic changes prior to study inclusion. Muscle tissue, fat and connective tissue were removed from all vertebrae. The specimens were stored at -20° C to preserve biomechanical properties for a longer period [5]. 24 h prior to study begin, the vertebrae were defrosted and frozen afterwards if needed.

### Study design

We had two hypotheses: Firstly, that cement-augmented pedicle screws had a higher pull-out strength in osteoprotic bone, compared to conventional pedicle screws, during primary surgery (= first test cycle). Secondly, that cement-augmented pedicle screws would demonstrate higher pull-out strength compared to conventional pedicle screws with a larger diameter during revision surgery in osteoporotic bone (= second test cycle).

All human vertebrae were prepared and then examined by dual-energy X-ray absorptiometry (DEXA) to assess bone quality. Study inclusion needed a density of bone under 0,8 g/cm^2^, which equals osteoporotic conditions.

**Fig. 1:**
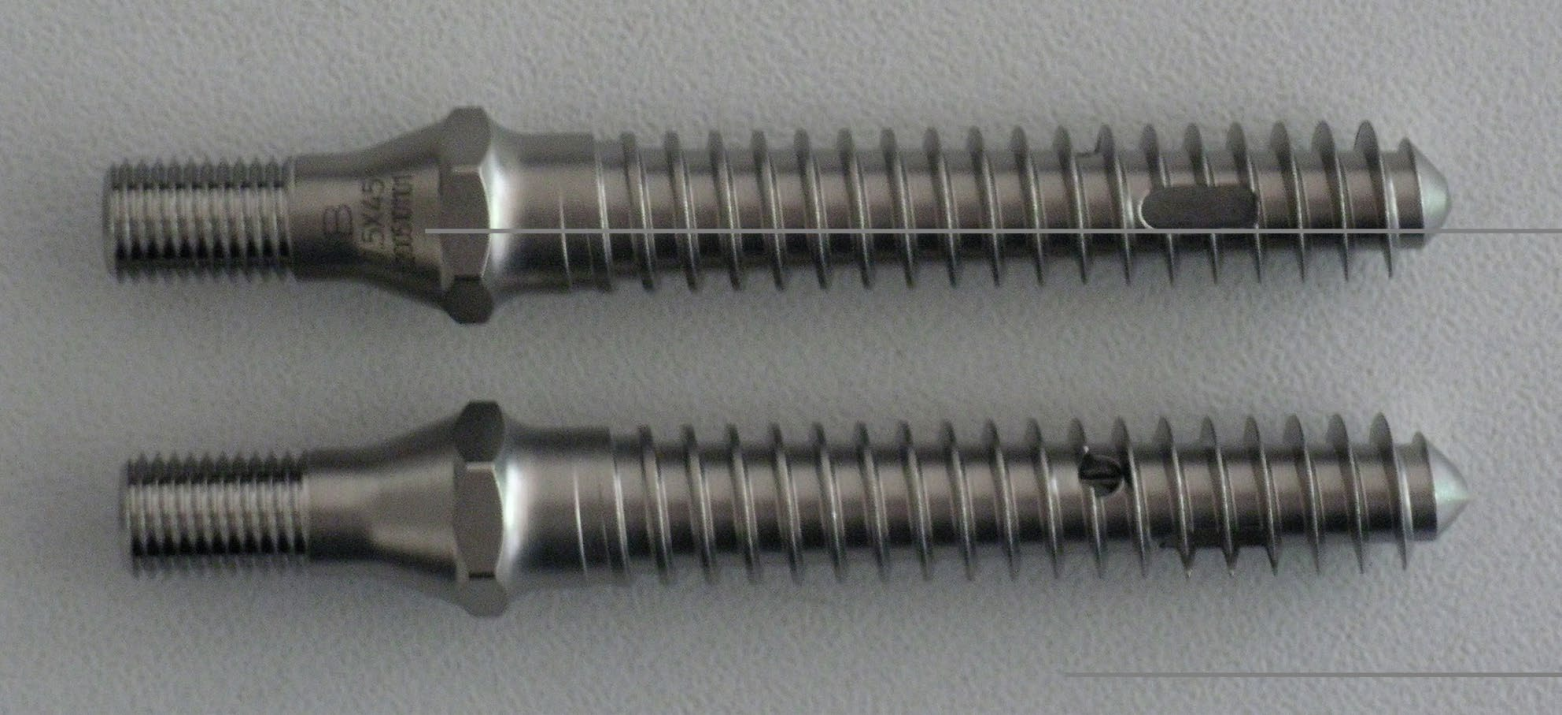
Vertebroplasty screw (VT, 6.35 mm x 45 mm, Zimmer Biomet, USA) with perforations for cement extravasation

Then primary, dorsal instrumentation was performed with two different pedicle screws, both by Zimmer Biomet (Warsaw, IN, USA)^®^:

1. a standard Omega^TM^-21 pedicle screw (6.35 mm x 45 mm) in 50 vertebrae (Group A).
2. a vertebroplasty screw (VT screw, 6.35 mm x 45 mm) in 15 Vertebrae (Group B). The VT screw is cannulated featuring a central drill hole at the tip. Two round (diameter of one pitch) and two oval side holes (two-pitch diameter) are positioned near the distal end of the screw, arranged 90° to each other to facilitate even cement extrusion (Fig. 1). Both screws had identical diameter, length and thread profile.

The pedicle screw entry point was defined as the intersection of two anatomical reference lines: a vertical line tangential to the lateral border of the superior articular process and a horizontal line passing through the center of the transverse (costal) process. From this point, the pedicle trajectory was prepared using a pedicle awl along the longitudinal axis. Screws were inserted with medial convergence and slight triangulation, with the pedicle itself determining the screw vector, following the technique described by Roy-Camille et al. [6]. Screw holes were neither tapped nor upsized in any of the experiments. The final screw position was assessed by CT scan.

For augmentation we used Osteopal^®^ V bone cement (Heraeus Medical, Germany). After mixing the two cement components and allowing a 2-minute waiting period to reach optimal viscosity (as recommended by the manufacturer), 2 ml of cement was injected under constant pressure using a Medtronic delivery system into the cannulated screw. Cement leakage and distribution were assessed intraoperatively by visual inspection and fluoroscopy, and postoperatively by CT in all vertebrae.

**Fig. 2:**
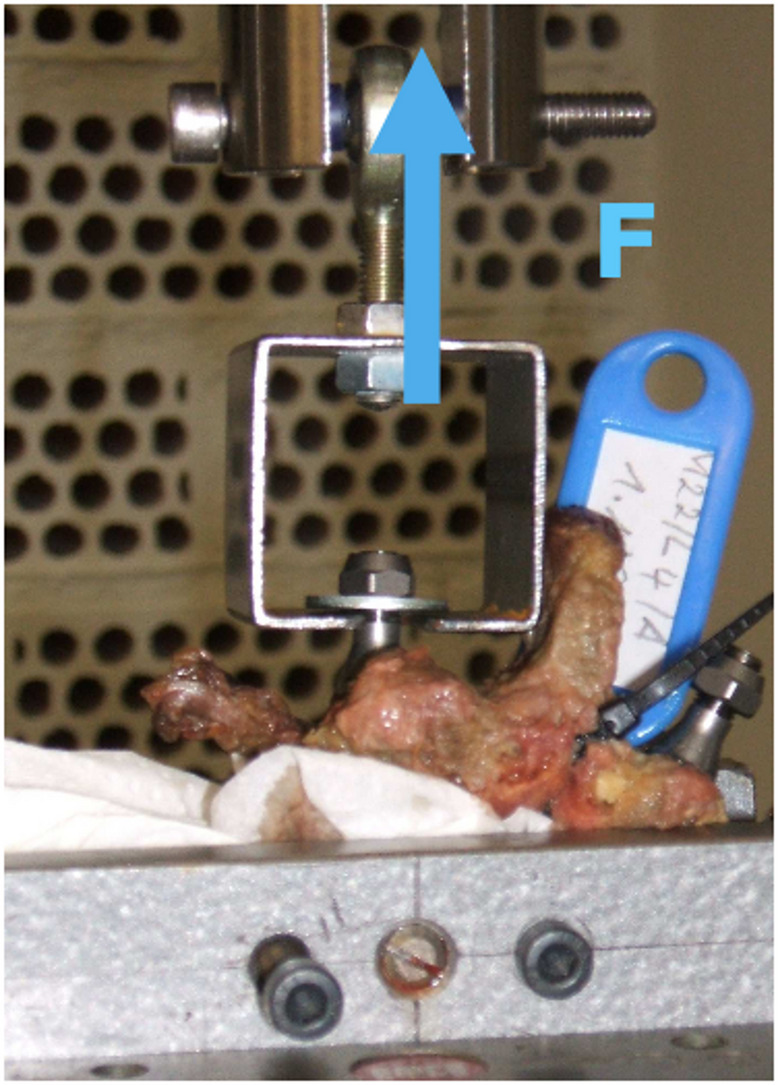
Vertebra Clamp and Load Direction

**Fig. 3:**
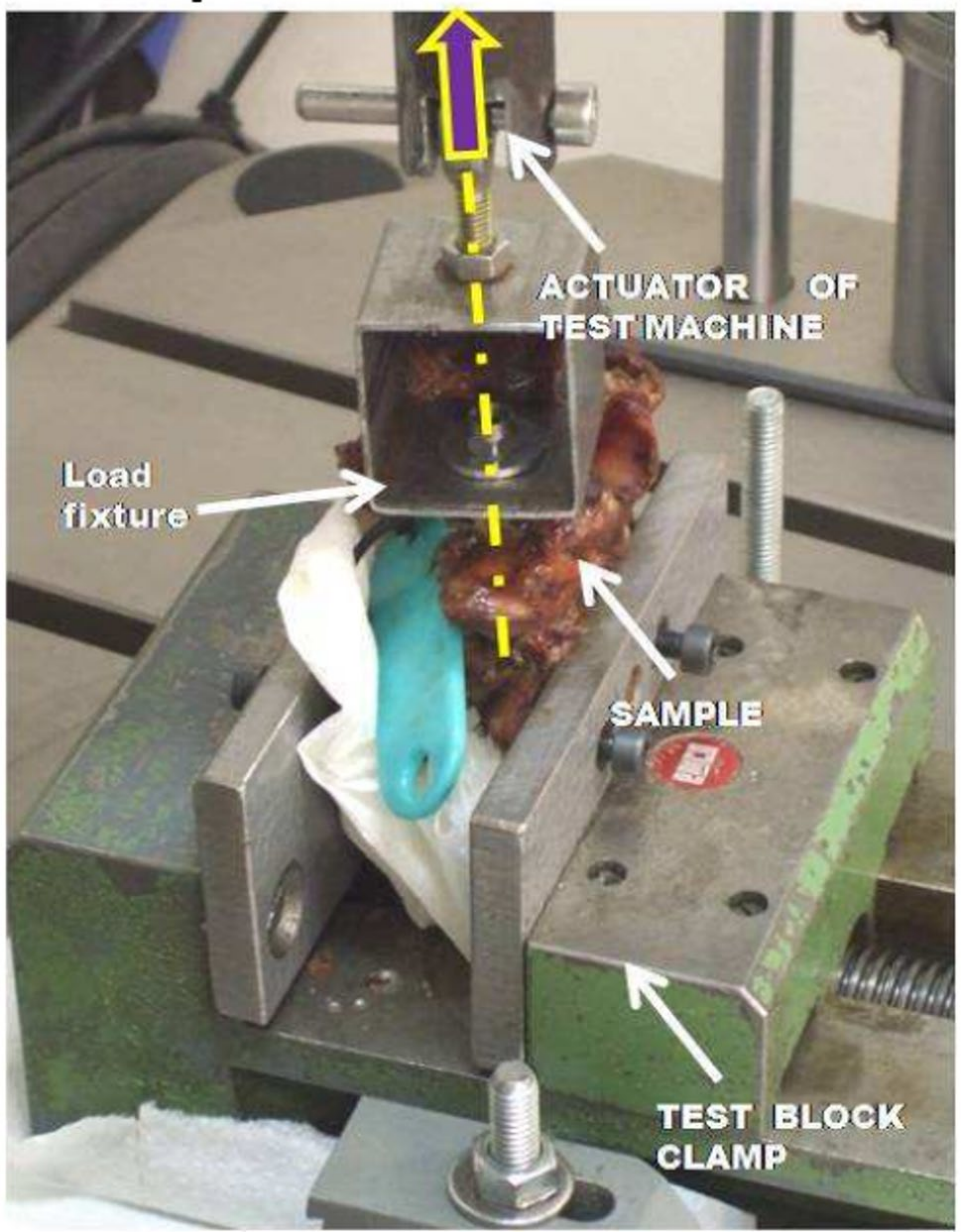
Vertebra Fixation and actuator

Specimens were stored at **− **20 °C and thawed 24 h prior to testing. The tests were conducted under ambient conditions of 22 °C temperature and *49% relative humidity*. For testing, each vertebra–screw assembly was mounted between the clamps of a *universal testing machine* (Instron 8874, West Conshohocken, PA, USA) (see Figs. 2 and 3) and a pull-out test cycle followed according to ASTM (American Standards of Testing Machines ) F543-07-standard [4], which were described before [7].

A tensile load was applied under displacement-controlled conditions at an actuator speed of 0.1 mm/s, and testing was terminated upon complete screw extraction.

The setup consisted of three components:

1. *Vertebra clamp* – used to secure the vertebra in the correct position. Proper alignment was confirmed when the screw’s longitudinal axis was parallel to the load direction (Fig. 2).
2. *Specimen *– composed of one vertebra with two transpedicular screws inserted.
3. *Testing machine (Instron 8874)* – equipped with a *5,000 N load cell*.

Figures 2 and 3 illustrate the schematic setup of the pull-out strength testing apparatus (in accordance with ASTM F543-07). Each vertebra with implanted pedicle screws was secured between the clamps (Figs. 2 and 3), with screw oriented vertically for the pull-out test (Fig. 2). Prior to testing, the actuator of the testing machine was aligned and fixed around the sample screw (Fig. 3).

**Fig. 4:**
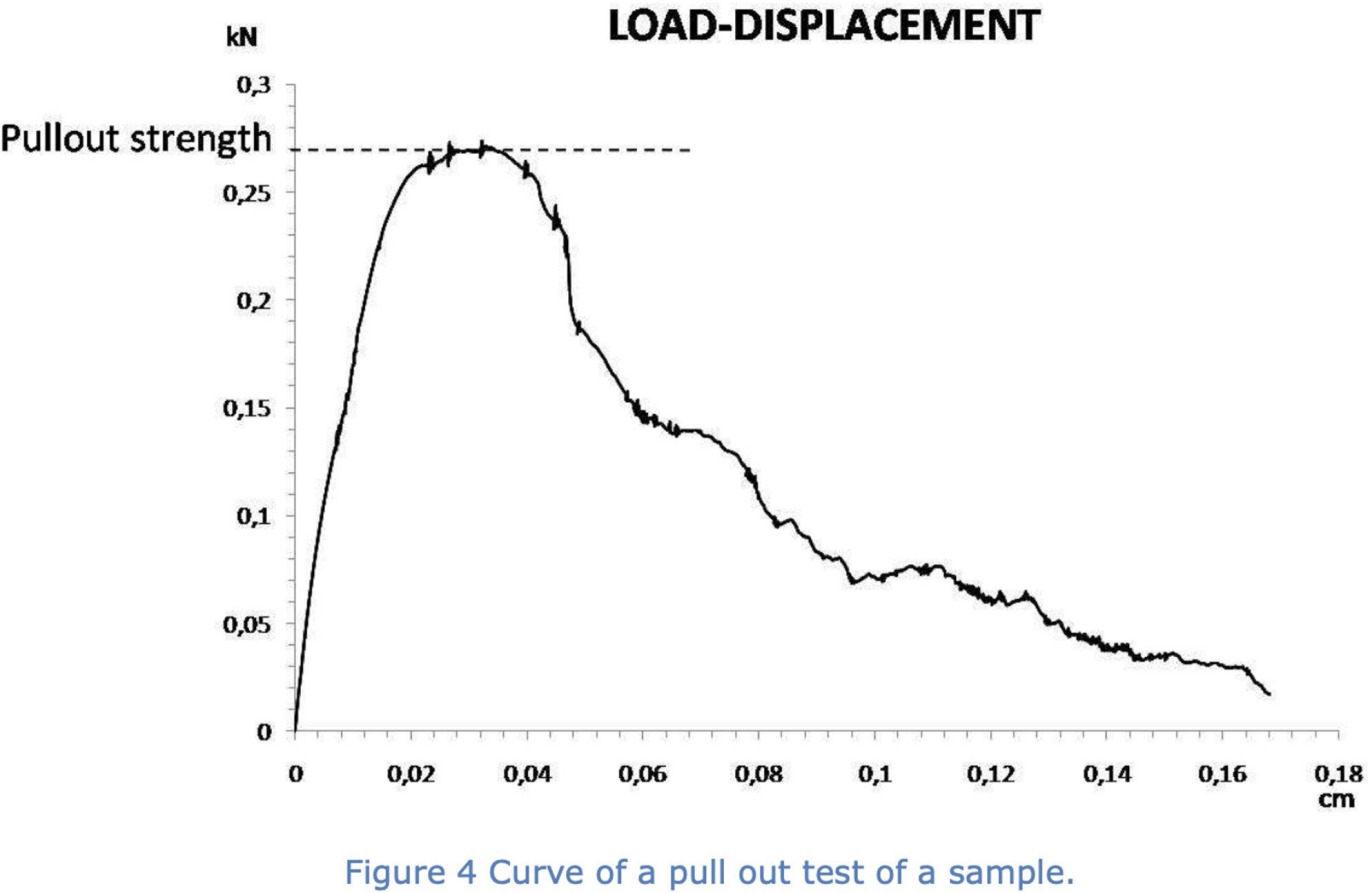
Curve of a typical pull-out load-displacement curve recorded during a pull-out test. The maximum force observed corresponds to the pull-out strength

The pull-out velocity was constant with 0.1 mm/second and pull forces were measured in the dynamometer between 0 and 5000 N. Pull-out tests were performed on each vertebra, first on the left and then on the right pedicle. A complete removal or a pull-out of more than 30 mm of the screws resulted in ending the test cycle. Failure mode was then defined as the sudden peak force at first drop. Figure 4 illustrates a load-displacement curve.

A standardized pull-out test was conducted on all 65 vertebrae (Cycle 1). Both pedicles of each vertebra were instrumented and subsequently tested under standardized conditions, resulting in two measurements per vertebra, yielding 100 measurements in Group A and 30 in Group B.

Following this first test cycle, all 15 vertebrae in the cement-augmented group (Group B) were structurally compromised and could not be included for further testing. The remaining 50 vertebrae, initially instrumented with conventional pedicle screws, exhibited pedicle damage consistent with conditions encountered in revision surgery.

These 50 vertebrae were subsequently reassigned to two groups (*n* = 25 each) for the second test cycle (Cycle 2). In Group A1, larger conventional pedicle screws (7.5 mm) were inserted in both pedicles. In Group A2, cement-augmented (VT) pedicle screws (6.35 mm) were used. A second pull-out test was then performed to evaluate pull-out strengths of both constructs. As in Cycle 1, both pedicles of each vertebra were tested, resulting in 50 measurements per group.

**Fig. 5:**
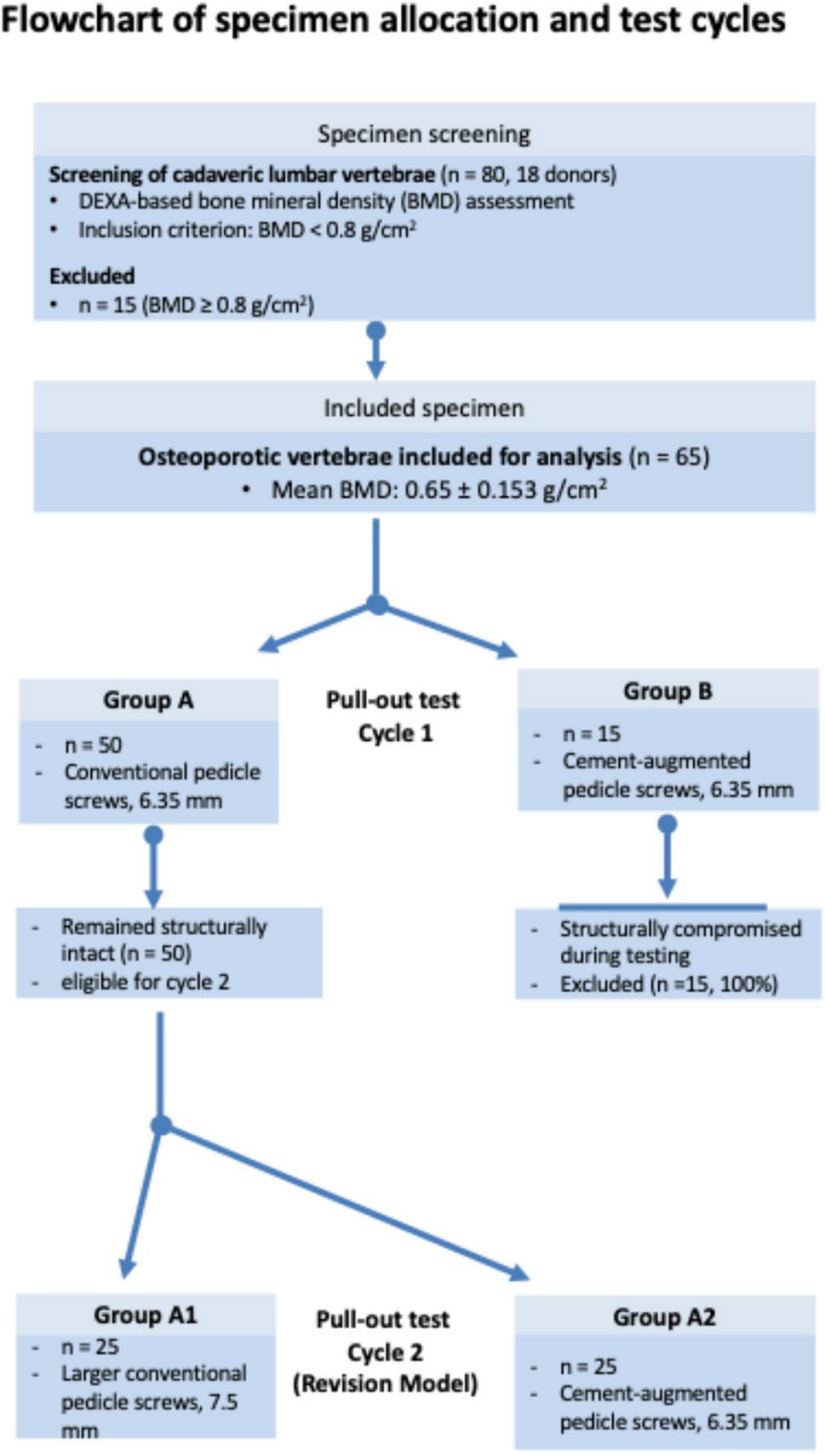
Flowchart of specimen screening, inclusion, allocation, and progression through both test cycles, including exclusions after Cycle 1

Specimen allocation and study flow are shown in Fig. 5.

### Statistics

Statistical analysis was performed using SigmaPlot^®^ 14.0 (Inpixon, Düsseldorf, Germany). Pull-out tests were conducted separately on each pedicle of all vertebrae. Normality was assessed using the Shapiro-Wilk test. Equality of variances was evaluated using the Brown-Forsythe test. If equal variances could not be assumed, Welch’s t-test was applied.

For normally distributed data with equal variances, parametric tests (paired or unpaired t-tests) and Pearson correlation were applied.

For non-normally distributed data, non-parametric tests (Wilcoxon signed-rank test or Wilcoxon rank-sum test) and Spearman’s rank correlation were used. Statistical significance was defined as *p* < 0.05 with a confidence interval (CI) of 95%.

Although group sizes in the first test cycle were unbalanced, the study design aimed to ensure sufficient statistical power. To evaluate potential bias due to group size imbalance, additional analyses including anomaly detection and random undersampling of the larger dataset were performed. As these analyses did not alter the results, the original full dataset was retained for final evaluation.

## Results

### Cadaveric specimens

A total of 80 lumbar bodies of eighteen donors, 8 women (44%) and 10 men (56%), were primarily prepared for the study. Mean age of the patients was 73 years (min. 63 - max. 97 years).

### Bone density

All 80 vertebrae underwent DEXA scanning. Osteoporosis was defined as a bone mineral density (BMD < 0.8 g/cm^2^. Overall, 81.25**% **(65/80) of the specimens exhibited a BMD < 0.8 g/cm², while the remaining 18.75% (15/80) showed values > 0.8 g/cm². Consequently, 65 lumbar vertebral bodies met the inclusion criteria, with a mean BMD of 0.65 ± 0.153 g/cm^2^ (range: 0.35–0.8 g/cm^2^).

No significant differences in BMD were observed between the study groups. Furthermore, no significant correlation was found between pull-out strength and bone density (Spearman: *p* = 0.0861, *r* = 0.261).

Intraclass correlation coefficients (ICC) between donors were consistently below 0.2, indicating low intra-donor correlation and supporting the assumption of independent observations.

All specimen in Group B (cement-augmented pedicle screws) were structurally compromised during the first test cycle and were excluded from further analysis. The remaining 50 vertebrae were used for the second test cycle and reassigned to Groups A1 and A2 (n= each). Matched-pair analysis was not feasible due to specimen loss after Cycle 1 and subsequent reallocation.

Although group sizes in the first test cycle were unbalanced, no bias affecting the results was detected. Additional analyses, including undersampling of the larger group, did not alter the outcomes.

### Cement distribution patterns

Cement distribution and potential leakage were assessed in all vertebrae of group B and A2. Intraoperatively, cement application was visually monitored and additionally evaluated using fluoroscopy. Postoperatively, all specimens underwent computed tomography (CT) for further assessment.

Macroscopic assessment during the experiment did not reveal cement leakage into segmental vessels (Type S) or the spinal canal (Type B, according to Yeom classification). A detailed analysis of cement distribution and leakage patterns will be presented in a separate study, with findings consistent with previously reported rates in the literature.

### Primary stabilization and first pull-out cycle (Cycle 1)

Group A (*n* = 50 specimens /100 measurements; 6.35 mm diameter conventional screws) demonstrated a mean pull-out strength of 662.8 ± 365.1 N (range: 14.4–1602 N). Group B (*n* = 15 specimens /30 measurements; 6.35 mm diameter cement-augmented screws) showed an average pull-out strength of 1066.1 ± 213.7 N (range: 200–1360 N).

**Fig. 6:**
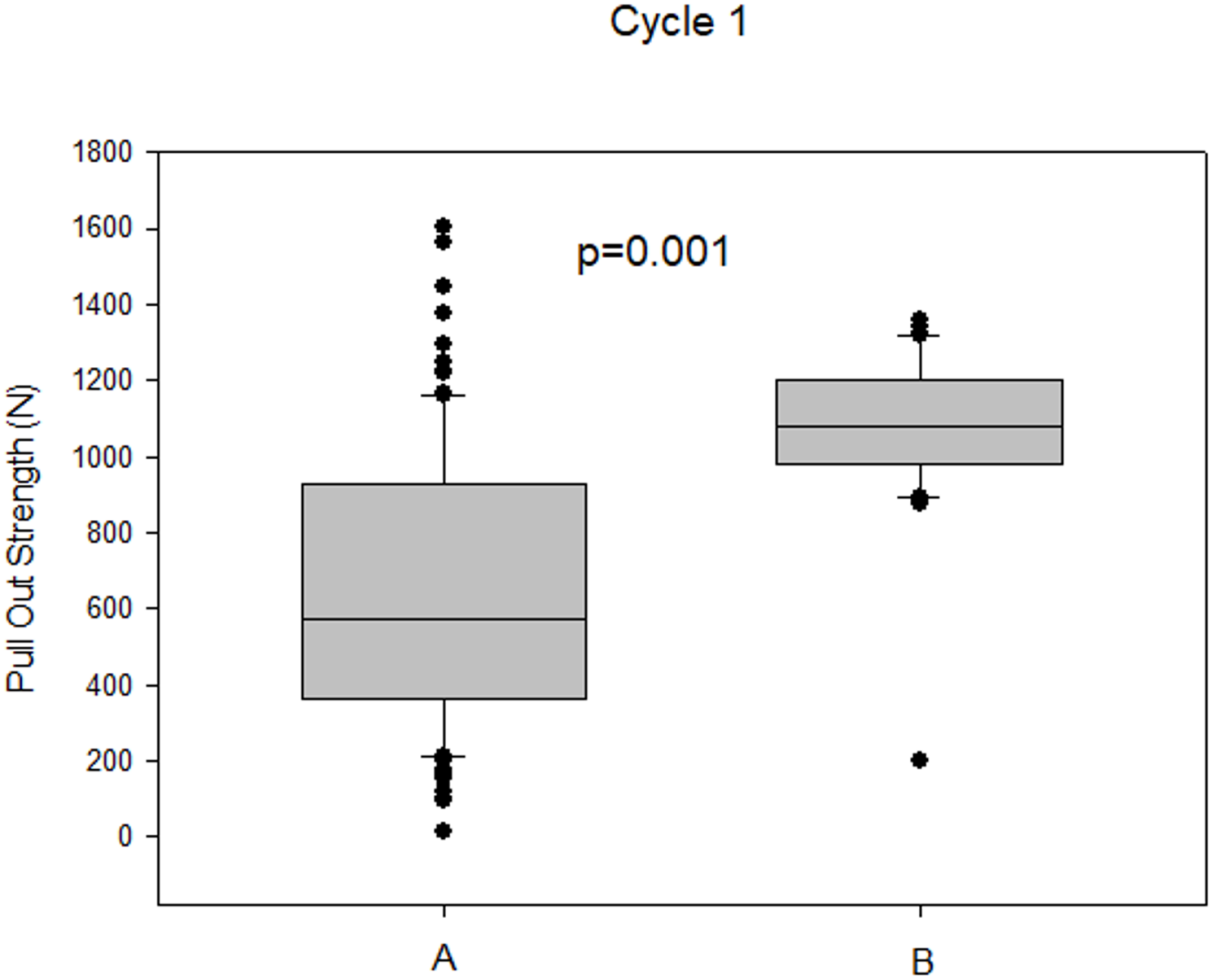
Comparison of pull-out strength between conventional pedicle screws (Group A, *n* = 50 specimens /100 measurements; 6.35 mm) and cement-augmented pedicle screws (Group B, *n* = 15 specimens /30 measurements; 6.35 mm) in osteoporotic bone (*p* < 0.001). Box plots display the median and inter-quartile range (25th – 75th percentile), with whiskers indicating the 5th and 95th percentiles. Individual data points are overlaid to illustrate data distribution. A higher number of extreme values was observed in Group A compared to Group B

Normal distribution of the data was confirmed (Shapiro-Wilk test, *p* = 0.325), whereas equality of variances was not assumed (Brown-Forsythe test, *p* < 0.05). Therefore, Welch’s t-test was applied, demonstrating a statistically significant difference between groups (mean difference: 403.2 N; 95% CI: 297.0–509.5 N; *p* < 0.001), with higher pull-out strength observed for cement-augmented screws (Fig. 6).

### Revision surgery and second pull-out cycle (Cycle 2)

Screws were inserted in the same vector as the loosened screws. No repositioning or tapping was performed.

Group A1 (*n* = 25 specimens/50 measurements; 7.5 mm diameter conventional screws) showed a median pull-out strength of 486.5 N [IQR: 338.8–605.0 N].

Group A2 (*n* = 25 specimens/50 measurements; 6.35 mm diameter cement-augmented pedicle screws) demonstrated a median pull-out strength of 687.5 N [IQR: 448.8–1137.5 N].

**Fig. 7:**
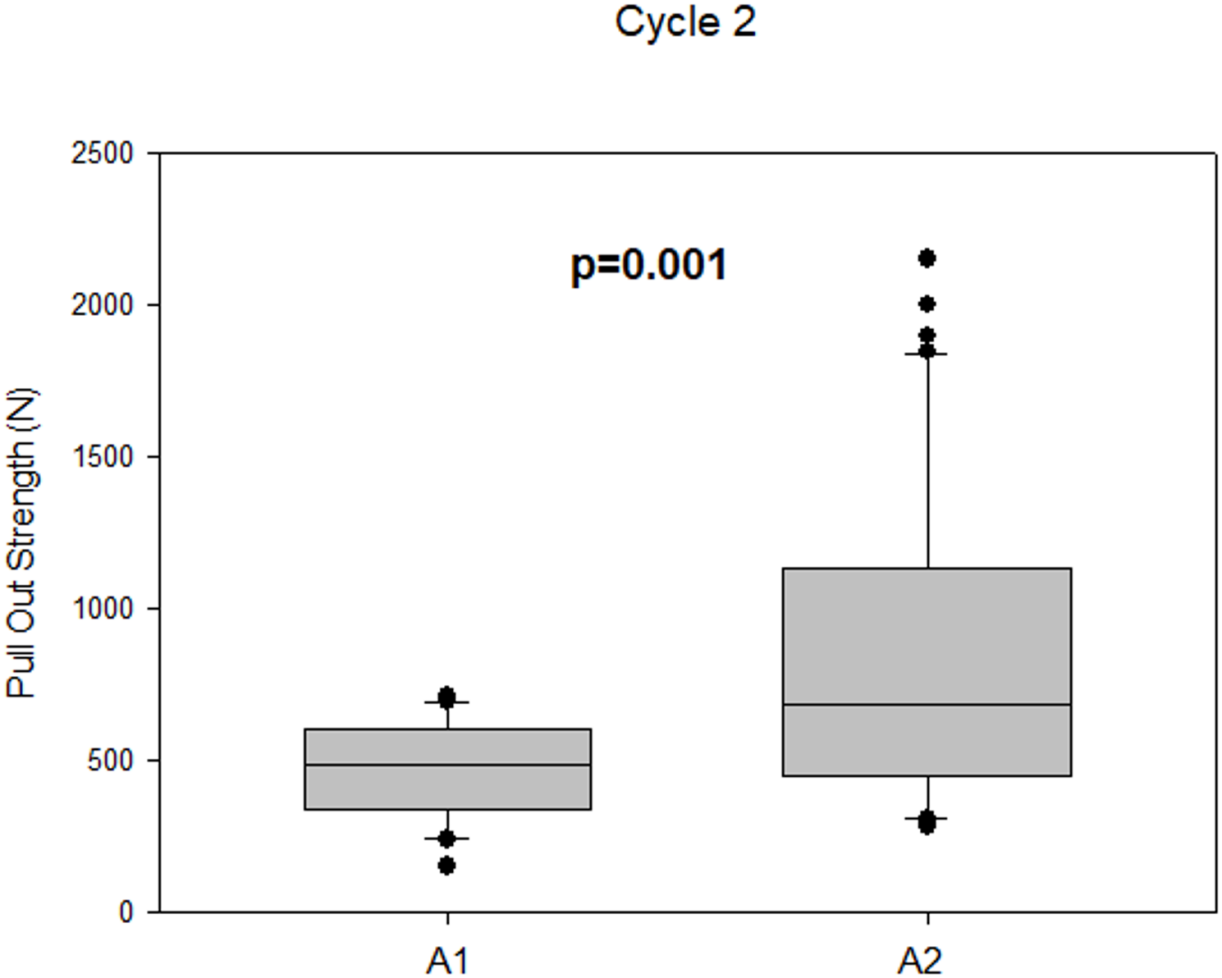
Comparison of pull-out strength after revision surgery using a larger conventional pedicle screws (Group A1, *n* = 25 specimens/50 measurements; 7.5 mm) and cement-augmented pedicle screws (Group A2, *n* = 25 specimens/50 measurements; 6.35 mm) in osteoporotic bone ( *p* = 0.001). Box plots display the median and inter-quartile range (25th – 75th percentile), with whiskers indicating the 5th and 95th percentiles. Individual data points are overlaid to illustrate data distribution

As the data were not normally distributed, a Mann-Whitney U test was applied. The differences between both study groups were statistically significant (U = 628.5, *p* = 0.001), indicating higher pull-out strength of cement-augmented pedicle screws (Group A2) compared to conventional screws (Fig. 7).

### Additional paired analysis

An additional paired analysis was performed comparing pedicle screws from Group A (*n* = 25 specimens; primary implantation) with cement-augmented pedicle screws in Group A2 (*n* = 25 identical specimens, reinstrumentation) resembling statistically dependent collectives. Median pull-out strength increased from 525.1 N [IQR: 313.6–712.5 N] in Group A to 687.5 N [IQR: 448.8–1137.5 N] in Group A2.

As the data were not normally distributed, a Wilcoxon signed-rank test was applied. The difference was statistically significant (W = 759.0, Z = 3.66, *p* < 0.001).

In contrast, a paired comparison between conventional pedicle screws used during primary instrumentation (Group A; 6.35 mm) and larger-diameter conventional screws used for reinstrumentation (Group A1; 7.5 mm) within the same specimens demonstrated a decrease in mean pull-out strength from 724.3 ± 365.5 N to 466.3 ± 165.2 N. This corresponds to a mean difference of -258.0 ± 383.0 N.

As normal distribution of the differences was confirmed (Shapiro-Wilk test, *p* = 0.698), a paired t-test was applied. The decrease in pull-out strength was statistically significant (t= -4.76, 95% CI: -366.8 to -149.2 N; *p* = 0.001).

The variability of pull-out strength was high across all groups. Inspection of the data distribution revealed a broad but continuous spread of values without evidence of extreme or implausible outliers. This variability reflects the heterogeneous mechanical properties of osteoporotic bone. All measurements were therefore retained for analysis. Individual data points are displayed in Figs. 6 and 7 to allow a detailed assessment of data distribution.

Overall, the results support both study hypotheses. In the first cycle (primary stabilization), cement-augmented pedicle screws demonstrated significantly higher pull-out strength compared to conventional screws. In the second test cycle (revision model), cement-augmented screws also showed superior pull-out strength compared to larger-diameter conventional screws. These findings were further reinforced by paired analyses within the same specimens, demonstrating consistently higher pull-out strength for cement-augmented screws in osteoporotic vertebrae.

## Discussion

Osteoporosis is a severe disease, which increases the risk for fracture of the femoral neck, radius and spine [8]. If spine surgery is indicated, the pedicle screws need to demonstrate a high primary stability. In case of revision spine surgery, osteoporotic conditions could cause screw loosening or further complications.

The aim of this study was to examine whether cement-augmented pedicle screws demonstrate superior biomechanical performance under conditions of low BMD. We found that cement-augmented screws had significantly higher stability compared to conventional screws. This superiority was found in both primary and revision surgery conditions ex vivo. These findings are in agreement with Bullmann et al. [9] who also found higher pull-out strength of augmented screws compared to conventional screws in a comparable experiment simulating primary surgery conditions with low BMD.

Bone loss and subsequent pedicle screw loosening represent a major clinical challenge. McLain et al. [10] suggested to replace a loosened 6 mm pedicle screw with an 8 mm pedicle, but not a 7 mm pedicle screw, as this had a higher pull-out strength. Another factor is the screw design. A long screw thread increases the bone-screw interface and therefore stability [11]. This is useful in revision. Cement can further improve the screw-bone interface by filling bony defects and providing higher pull-out strength [11].

Previous studies were able to demonstrate the superiority of augmented screws and only low complication rates [7, 12]. Perforated pedicle screws are superior compared to conventional screws combined with a vertebroplasty [13]. In many cases, and in our study, PMMA (polymethylmethacrylate)-based cement is used for augmentation. Alternatively, calciumphosphate cement can be used. Both cement forms can increase vertebral stiffness [1]. Several authors established a correlation between a low bone mineral density and screw pull-out strength [14–16].

The anatomy of the pedicle and the distribution of cancellous and cortical bone is still discussed. One study found a central cancellous tissue and a thick cortical bone circumference [6].

Theoretically, this would allow a stable anchoring of the screw in the outer limits of the pedicle. Contrary, Hirano et al. [17] and Misenhimer et al. [18] described a rather thin cortex of the pedicle and that the pedicle screw would grab tight in the thicker cancellous bone.

In osteoporosis the quantity of both, cancellous and cortical bone can be reduced by almost 50% [17, 19]. Stability of pedicle screws decreases by almost 75% in correlation to osteopenic bone stages [20]. The benefit of larger diameters of pedicle screws with higher pull-out strength was well described in normal bone [13]. In osteoporotic conditions this benefit is significantly reduced [9, 17].

The augmentation of the cancellous, osteoporotic bone with cement ensured a tight anchoring of the screws, compared to the use of larger diameters of conventional screws [9]. A possible explanation is that the cement increases contact surface around the screw and stiffness in the bone [21]. The applied amount of PMMA-based cement should not exceed 4–5 ml per vertebrae [22].

Despite these biomechanical advantages, a considerable variability in pull-out strength was observed in this study.

Across the two test cycles, the distribution of the data differed. In the first cycle, the data were normally distributed, whereas in the second cycle a non-normal distribution was assessed, reflecting increased heterogeneity under revision conditions. The broader spread of values, particularly in the revision model, likely reflects the heterogeneous mechanical properties of osteoporotic bone in our specimen cohort, including local differences in trabecular architecture and bone quality, as previously described [23, 24]. Despite this variability, cement-augmented pedicle screws demonstrated significantly higher pull-out strength under both primary and revision conditions.

Importantly, the additional paired analyses performed within the same specimens provided additional support for theses findings. By minimizing inter-specimen variability, this approach consistently confirmed the observed differences between fixation strategies under both primary and revision conditions, thereby providing robust internal validation of the results.

This study has limitations. First, we used an ex vivo model with dynamometer and frozen vertebral specimens. This cadaveric model has been described previously [4], but still does not fully resemble complex, cyclic and multi-directional in vivo loading conditions. Therefore, the results of this study should be interpreted with caution when extrapolating to clinical scenarios.

Axial pull-out tests for evaluation of the pedicle screw anchorage are easy to conduct and give a practical parameter, but it must also be considered that they do not reflect the clinical setting [11, 13, 25] as they apply an unphysiological loading to the pedicle screw. The characteristic “windshield-wiper effect”, typically observed under cyclic loading conditions in clinical practice, cannot be reproduced in a purely axial pull-out model [26].

In this study, we used pull-out testing. Pull-out is considered the standard testing method, as there is a vast historical database of test results, it is considered a relatively easy and consistent test to perform, and a standardized testing method is established (ASTM F543) [27]. Although comparisons with other studies are easier, clinical applicability is to be tested eventually under cyclic loading conditions. Effects of muscle and connective tissue, body weight and size were not taken into account. Long-term effects of screw loosening etc. cannot be explored.

Second, in vivo experiments in animals could demonstrate further findings, but are not fully comparable due to the quadruped standing and therefore different anatomic conditions and strains compared to a human spine.

Third, we present data of a rather small sample size of 50 human cadaveric vertebrae (Group A; 100 measurements) and 25 vertebrae (50 measurements) for subgroups A1 and A2. This is still more than most of test cycles reported in the literature, but a larger cohort could further explore the results, although this is cost-intensive.

Randomized controlled trials should further investigate the benefits of these findings to ensure secure and stable dorsal instrumentation in patients.

Based on the authors’ clinical experience, current spinal revision procedures, particularly in osteoporotic bone, primarily involve the use of larger screws, bolts or similar fixation devices or even extension of the construct sometimes inducing proximal junctional kyphosis. Although the advantages of cement-augmented pedicle screws have been previously described, their clinical application warrants more extensive discussion. We therefore consider an updated evaluation of this topic to be highly relevant, as our findings reinforce the potential benefits of cement-augmented pedicle screws, particularly in light of the anticipated increase in spinal revision surgeries and osteoporotic cases in the coming years.

## Conclusion

In summary, this study demonstrated that cement-augmented pedicle screws provide significantly higher pull-out strength compared to conventional screws under both primary and revision conditions in an ex vivo model of osteoporotic bone. Further clinical investigation is required. Randomized controlled trials are needed to evaluate the clinical relevance and long-term outcomes of cement-augmented pedicle screws in osteoporotic patients.

## Data Availability

For reasons of data protection and data volume, the raw data cannot be made publicly available. However, relevant data extracts or aggregated data can be provided upon justified request from the corresponding author.

## References

[1] Mills ES, Ton AT, Bouz G, Alluri RK, Hah RJ. Acute Operative Management of Osteoporotic Vertebral Compression Fractures Is Associated with Decreased Morbidity. Asian Spine J. 2022;16:634–42. 10.31616/asj.2021.0297. 35184517 10.31616/asj.2021.0297 PMC9633232

[2] Althoff AD, Kamalapathy P, Vatani J, Hassanzadeh H, Li X. Osteoporosis is associated with increased minor complications following single level ALIF and PSIF: an analysis of 7,004 patients. J Spine Surg. 2021;7:269–76. 10.21037/jss-21-29. 34734131 10.21037/jss-21-29 PMC8511573

[3] Puvanesarajah V, Shen FH, Cancienne JM, Novicoff WM, Jain A, Shimer AL, Hassanzadeh H. Risk factors for revision surgery following primary adult spinal deformity surgery in patients 65 years and older. J Neurosurg Spine. 2016;25:486–93. 10.3171/2016.2.SPINE151345. 27153147 10.3171/2016.2.SPINE151345

[4] Wilke HJ, Wenger K, Claes L. Testing criteria for spinal implants: recommendations for the standardization of in vitro stability testing of spinal implants. Eur Spine J. 1998;7:148–54. 10.1007/s005860050045. 9629939 10.1007/s005860050045 PMC3611233

[5] Panjabi MM, Krag M, Summers D, Videman T. Biomechanical time-tolerance of fresh cadaveric human spine specimens. J Orthop Res. 1985;3:292–300. 10.1002/jor.1100030305. 4032102 10.1002/jor.1100030305

[6] Roy-Camille R, Saillant G, Mazel C. (1986) Internal fixation of the lumbar spine with pedicle screw plating. Clin Orthop Relat Res:7–17. 3955999

[7] Wichmann JL, Booz C, Wesarg S, Bauer RW, Kerl JM, Fischer S, Lehnert T, Vogl TJ, Khan MF, Kafchitsas K. Quantitative dual-energy CT for phantomless evaluation of cancellous bone mineral density of the vertebral pedicle: correlation with pedicle screw pull-out strength. Eur Radiol. 2015;25:1714–20. 10.1007/s00330-014-3529-7. 25481639 10.1007/s00330-014-3529-7

[8] Kanis JA, Harvey NC, McCloskey E, Bruyere O, Veronese N, Lorentzon M, Cooper C, Rizzoli R, Adib G, Al-Daghri N, Campusano C, Chandran M, Dawson-Hughes B, Javaid K, Jiwa F, Johansson H, Lee JK, Liu E, Messina D, Mkinsi O, Pinto D, Prieto-Alhambra D, Saag K, Xia W, Zakraoui L, Reginster JY. Correction to: Algorithm for the management of patients at low, high and very high risk of osteoporotic fractures. Osteoporos Int. 2020;31:797–8. 10.1007/s00198-020-05297-0. 32065251 10.1007/s00198-020-05297-0 PMC7075819

[9] Bullmann V, Schmoelz W, Richter M, Grathwohl C, Schulte TL. Revision of cannulated and perforated cement-augmented pedicle screws: a biomechanical study in human cadavers. Spine (Phila Pa 1976). 2010;35:E932–939. 10.1097/BRS.0b013e3181c6ec60. 20508553 10.1097/BRS.0b013e3181c6ec60

[10] McLain RF, Fry MF, Moseley TA, Sharkey NA. Lumbar pedicle screw salvage: pullout testing of three different pedicle screw designs. J Spinal Disord. 1995;8:62–8. 7711371

[11] Zindrick MR, Wiltse LL, Widell EH, Thomas JC, Holland WR, Field BT, Spencer CW. (1986) A biomechanical study of intrapeduncular screw fixation in the lumbosacral spine. Clin Orthop Relat Res:99–112. 3956001

[12] Kafchitsas K, Geiger F, Rauschmann M, Schmidt S. [Cement distribution in vertebroplasty pedicle screws with different designs]. Orthopade. 2010;39:679–86. 10.1007/s00132-010-1603-7. 20549485 10.1007/s00132-010-1603-7

[13] Kueny RA, Kolb JP, Lehmann W, Puschel K, Morlock MM, Huber G. Influence of the screw augmentation technique and a diameter increase on pedicle screw fixation in the osteoporotic spine: pullout versus fatigue testing. Eur Spine J. 2014;23:2196–202. 10.1007/s00586-014-3476-7. 25082759 10.1007/s00586-014-3476-7

[14] Jacob AT, Ingalhalikar AV, Morgan JH, Channon S, Lim TH, Torner JC, Hitchon PW. Biomechanical comparison of single- and dual-lead pedicle screws in cadaveric spine. J Neurosurg Spine. 2008;8:52–7. 10.3171/SPI-08/01/052. 18173347 10.3171/SPI-08/01/052

[15] Halvorson TL, Kelley LA, Thomas KA, Whitecloud TS 3rd, Cook SD. Effects of bone mineral density on pedicle screw fixation. Spine (Phila Pa 1976). 1994;19:2415–20. 10.1097/00007632-199411000-00008. 7846594 10.1097/00007632-199411000-00008

[16] Cook SD, Salkeld SL, Stanley T, Faciane A, Miller SD. Biomechanical study of pedicle screw fixation in severely osteoporotic bone. Spine J. 2004;4:402–8. 10.1016/j.spinee.2003.11.010. 15246300 10.1016/j.spinee.2003.11.010

[17] Hirano T, Hasegawa K, Takahashi HE, Uchiyama S, Hara T, Washio T, Sugiura T, Yokaichiya M, Ikeda M. Structural characteristics of the pedicle and its role in screw stability. Spine (Phila Pa 1976). 1997;22:2504–9. 10.1097/00007632-199711010-00007. discussion 2510. 9383856 10.1097/00007632-199711010-00007

[18] Misenhimer GR, Peek RD, Wiltse LL, Rothman SL, Widell EH Jr. Anatomic analysis of pedicle cortical and cancellous diameter as related to screw size. Spine (Phila Pa 1976). 1989;14:367–72. 10.1097/00007632-198904000-00004. 2718038 10.1097/00007632-198904000-00004

[19] Becker S, Chavanne A, Spitaler R, Kropik K, Aigner N, Ogon M, Redl H. Assessment of different screw augmentation techniques and screw designs in osteoporotic spines. Eur Spine J. 2008;17:1462–9. 10.1007/s00586-008-0769-8. 18781342 10.1007/s00586-008-0769-8 PMC2583182

[20] Soshi S, Shiba R, Kondo H, Murota K. An experimental study on transpedicular screw fixation in relation to osteoporosis of the lumbar spine. Spine (Phila Pa 1976). 1991;16:1335–41. 10.1097/00007632-199111000-00015. 1750009 10.1097/00007632-199111000-00015

[21] Hsieh MK, Chen WP, Lee DM, Li YD, Kao FC, Chiang HH, Tsai TT, Fu TS, Lai PL, Tai CL. The biomechanical impact of cement volume and filling pattern for augmented pedicle screws using various density testing blocks. Spine J. 2025;25:1542–51. 10.1016/j.spinee.2025.01.018. 39894279 10.1016/j.spinee.2025.01.018

[22] Liebschner MA, Rosenberg WS, Keaveny TM. Effects of bone cement volume and distribution on vertebral stiffness after vertebroplasty. Spine (Phila Pa 1976). 2001;26:1547–54. 10.1097/00007632-200107150-00009. 11462084 10.1097/00007632-200107150-00009

[23] Varghese V, Kumar GS, Krishnan V. Effect of various factors on pull out strength of pedicle screw in normal and osteoporotic cancellous bone models. Med Eng Phys Feb. 2017;40:28–38. 10.1016/j.medengphy.2016.11.012 27939099

[24] Hollensteiner M, Greinwald M, Sandriesser S, Augat P. Screw pullout strength in osteoporotic bone: A comparative study of synthetic femur surrogates. J Biomech 2025 Sep. 2025;190:112880. 10.1016/. 10.1016/j.jbiomech.2025.112880 40706324

[25] Law M, Tencer AF, Anderson PA. Caudo-cephalad loading of pedicle screws: mechanisms of loosening and methods of augmentation. Spine (Phila Pa 1976). 1993;18:2438–43. 10.1097/00007632-199312000-00012. 8303446 10.1097/00007632-199312000-00012

[26] Bostelmann R, Keiler A, Steiger HJ, et al. Effect of augmentation techniques on the failure of pedicle screws under cranio-caudal cyclic loading. Eur Spine J. 2017;26:181–8. 10.1007/s00586-015-3904-3. 25813011 10.1007/s00586-015-3904-3

[27] Standard Specification and Test Methods for Metallic. Medical Bone Screws. https://store.astm.org/f0543-17.html.

